# Immune cells in liver regeneration

**DOI:** 10.18632/oncotarget.12275

**Published:** 2016-09-27

**Authors:** Na Li, Jinlian Hua

**Affiliations:** ^1^ College of Veterinary Medicine, Shaanxi Center of Stem Cells Engineering & Technology, Northwest A&F University, Yangling, Shaanxi, China

**Keywords:** liver, partial hepatectomy, regeneration, innate immune system, adaptive immune system

## Abstract

After partial hepatectomy, hepatocytes proliferate to restore mass and function of the liver. Macrophages, natural killer (NK) cells, natural killer T (NKT) cells, dendritic cells (DC), eosinophils, gamma delta T (?dT) cells, and conventional T cells, as well as other subsets of the immune cells residing in the liver control liver regeneration, either through direct interactions with hepatocytes or indirectly by releasing inflammatory cytokines. Here, we review recent progress regarding the immune cells in the liver and their functions during liver regeneration.

## INTRODUCTION

The liver is the largest solid organ within the body. It receives about 20% of its blood supply from the hepatic artery and 80% from the portal vein [1–3]. Owing to its anatomical location and blood supply, the liver is the first line of defense against various blood-borne pathogens and is very important to host immunity and survival [3–6]. To accomplish its immunological roles, the liver is enriched in several subsets of innate (such as macrophages, NK cells, NKT cells, neutrophils, γδT cells, dendritic cells, innate lymphoid cells) [6–8] and adaptive immune cells (such as T cells and B cells) [3, 9], which are finely tuned to affect the status of immuno-tolerance, pathogen clearance, tumor progression, and acute injury of the liver (refer to Table 1 for the information of non-parenchymal cell subsets in the liver) [3, 6, 7, 10].

**Table 1:** The non-parenchymal cell subsets in the liver

Cell subsets		Markers	Percentage
Non-immune cells (CD45-)	Sinusoidal endothelial cell [132]	VEGFR2+VEGFR3+VE-cadherin+CD31+CD34-	50%
	Cholangiocyte [3]	Cytokeratin-7, cytokeratin-19	5%
	Stellate cell [3]	Quiescent: desmin, GFAP Activated: α-SMA	<1%
Immune cells (CD45+)	Macrophage [9, 133]	Resident: F4/80highCD11blow Circulating: F4/80lowCD11bhigh	20%
	NK [9, 133]	CD3-NK1.1+/DX5+	6%
	NKT [9, 133]	CD3+CD1dTetramer+	8%
	αβT [8]	CD3+NK1.1-	6.5%
	γδT [6, 74]	CD3+TCRγδ+	1.5%
	B [8]	CD19+	2%
	Others [8]	--	1%

Abbreviations: VEGFR, vascular endothelial growth factor receptor; GFAP: glial fibrillary acidic protein; α-SMA, alpha-smooth muscle actin.

Normally quiescent hepatocytes will undergo proliferation in response to various stimulations, such as toxic injury, viral infection and surgery. Most studies concerning liver regeneration take the advantage of the two-thirds partial hepatectomy model in mice or rats. In this model, two-thirds of the liver, usually the median and left lateral lobes, is surgically removed. In response to this, the remnant liver enlarges until it restores normal mass and functions [11–13]. This process usually takes about 10 days, after which the regeneration process stops. Unlike the conventional meaning of ‘regeneration’, which usually means the complete re-growth of an excised tissue [14], liver regeneration is a very different process, which does not lead to the restoration of the excised lobules, but rather the compensatory hyperplasia of the remnant lobules.

**Figure 1:**
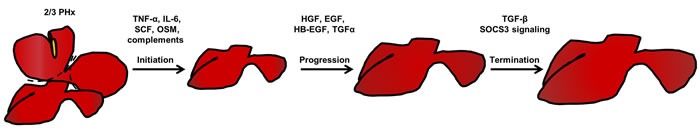
Three phases of liver regeneration after 2/3 partial hepatectomy After 2/3 partial hepatectomy, an acute phase response initiates the liver regeneration process. In this process, the complement system in the liver is activated, which triggers different cytokines needed for regeneration priming. Among these cytokines, TNF-α and IL-6 are the most important. In addition, SCF and OSM are beneficial for enhancing the effects of these regeneration-promoting cytokines. In response to this, quiescent hepatocytes enter the cell cycle (Go to G1 phase). Then, various growth factors, such as HGF, EGF, HB-EGF, and TGF-α further drive the cell cycle to S phase, which is the progression phase of liver regeneration. When the liver re-establishes its normal mass and function, signals terminating the regeneration process, such as TGF-β and SOCS3 signals, brakes the regeneration process, and the liver accomplishes the regeneration process after 2/3 partial hepatectomy. Abbreviations: TNF-α, tumor necrosis factor-α; IL-6, interlukin-6; SCF, stem cell factor; OSM, Oncostatin M; HGF, hepatocyte growth factor; EGF, epidermal growth factor; HB-EGF, heparin-binding epidermal growth factor; TGF-α, transforming growth factor-α; TGF-β, transforming growth factor-β; SOCS3, Suppressor Of Cytokine Signaling 3.

There have been different groups of researchers attempting to explain the mechanisms of liver regeneration. Accumulating evidence demonstrates that partial hepatectomy can lead to an acute phase response in the liver, during which the immune system will be robustly activated, and inflammatory mediators, including cytokines, chemokines, and complements will be released, stimulating quiescent hepatocytes to enter the G1 phase of cell cycle. Thereafter, various growth factors are secreted to further enhance the proliferation of the primed hepatocytes. At last, inhibiting signals are activated to avoid excessive regeneration, until the liver restores its normal mass, architecture, and function (Figure 1) [11, 12]. The effects of these mediators are complicated and finely tuned to ensure an efficient and effective regeneration process. Here, we mainly summarize the recent literatures concerning the immune system in the liver and their functions during the process of liver regeneration.

## THE INNATE IMMUNE SYSTEM AND LIVER REGENERATION

### Macrophages in liver regeneration

It was formerly believed that all macrophages were differentiated from blood monocytes [15, 16]. However, only recently did researchers find that there were in fact two distinct populations of macrophages in various tissues according to their progenitors and development process, namely yolk-sac-derived tissue-resident macrophages and bone marrow-derived circulating macrophages. The former were F4/80highCD11blow and the latter were CD11bhighF4/80low in various tissues [17, 18]. In fact, tissue-resident macrophages and bone marrow-derived macrophages have been demonstrated to play distinct and non-redundant roles in models of injury, repair, and regeneration [19–24].

In response to inflammatory signals, macrophages could be polarized into two functionally distinct subsets, namely M1 and M2 macrophages. Interferon-γ (IFN-γ) and LPS lead to the M1 activation of macrophages (classical activation), whereas IL-4 and IL-13 induce the M2 activation of macrophages (alternative activation) [25–28]. The M1 phenotype is exemplified by high levels of pro-inflammatory cytokines, high secretion of reactive oxygen and nitrogen intermediates, which promote strong tumoricidal and microbicidal activities. On the other hand, M2 activation is characterized by strong phagocytic activity, high production of ornithine and polyamines and expression of mannose, scavenging, and galactose receptors. M2 macrophages mainly exert protumoral and immunoregulatory functions [29–31].

**Figure 2:**
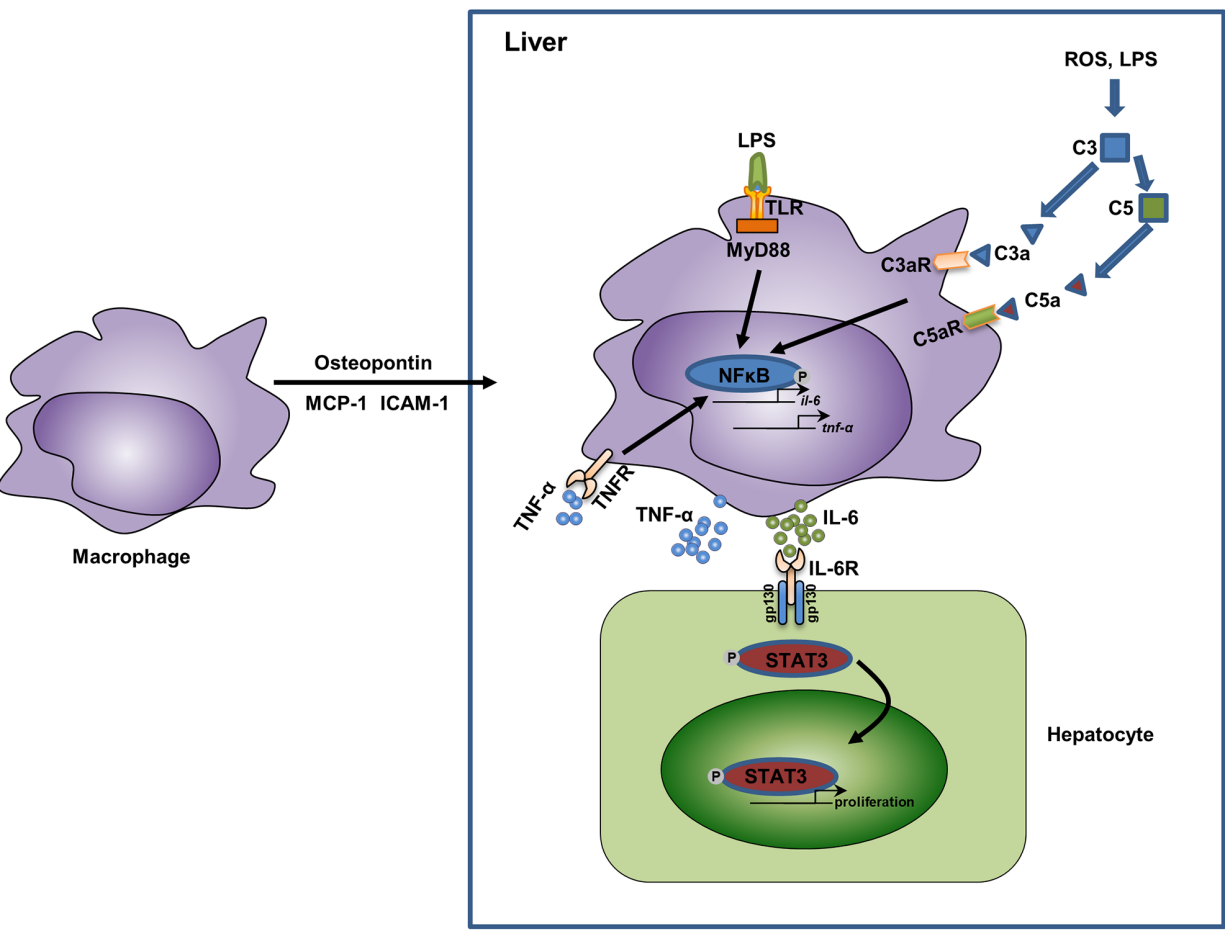
The relationship of macrophage activation, cytokine secretion, and liver regeneration In response to partial hepatectomy, the liver shows an acute phase response, during which the liver is enriched in several chemotaxis mediators for macrophages, such as osteopontin (mainly secreted by biliary epithelial cells [47]), MCP-1 (mainly secreted by hepatic stellate cells and biliary epithelial cells [130, 131] ), and ICAM-1 (mainly secreted by sinusoidal endothelial cells [40]). In the liver, the increased levels of LPS as well as reactive oxygen species (ROS) activate the complement system and the TLR/MyD88 pathway in macrophages. This leads to the activation of NFκB and results in the release of inflammatory cytokines TNF-α and IL-6. TNF-α could function in an autocrine manner and further activate NFκB. IL-6 binds to its receptors on hepatocytes and activate STAT3 signaling, thus promote the proliferation of hepatocytes. Abbreviations: MCP-1, monocyte chemoattractant protein-1; ICAM-1, intercellular adhesion molecule 1; ROS, reactive oxygen species; TNF-α, tumor necrosis factor-α; IL-6, interlukin-6; STAT3, Signal transducer and activator of transcription 3.

In the liver, macrophages represent about 20% of the non-parenchymal cells. They serve as the immune sentinel of the liver, sensing various stimulants and alerting other immune cells through delicate cell-cell interaction and secreted cytokines [3, 32]. Among the innate immune system in the liver, macrophages are the most extensively studied cells during liver regeneration. Several lines of evidence demonstrated that macrophage activation is beneficial to liver regeneration and provide the initial priming force for hepatocyte proliferation. The most convincing evidence would be experiments involved in macrophage depletion. A number of groups found that macrophage depletion would greatly compromise the regeneration rate of the liver [33–36]. It is believed that macrophage-derived cytokines such as tumor necrosis factor-α (TNF-α) and IL-6 were the most important forces for liver regeneration after partial hepatectomy [12, 37–39]. The results of several different groups corroborated this notion. For example, macrophage-depleted mice failed to exert a similar cytokine response thus resulted in retarded liver regeneration after partial hepatectomy [33, 40–42]. And macrophage colony stimulating factor (M-CSF) deficiency was reported to impair liver regeneration [43]. To elucidate the mechanism in this, it was shown that the complement components C3a and C5a were critical for activating macrophages through complement receptors, and stimulated the production of IL-6 and TNF-α, thus primed liver regeneration after partial hepatectomy [44]. Besides, it was indicated that TLRs/MyD88 signaling was responsible for the production of TNF-α and IL-6 from Kupffer cells [45]. The upregulation of these cytokines in response to partial hepatectomy was suggested to be due to the stimulation of liver macrophages by enteric-derived bacterial products [12, 42, 46]. In addition, monocyte chemoattractant protein-1 (MCP-1), intercellular adhesion molecule 1 (ICAM-1), and osteopontin were demonstrated to be the factors responsible for recruiting macrophages to the liver after partial hepatectomy (Figure 2) [34, 40, 47].

Besides these directly mitogenic functions, liver macrophages could also interact with other cell subsets thus indirectly promote the process of liver regeneration. Recently, it was reported that gut commensal microbiota, especially ampicillin sensitive bacteria were essential to keep Kupffer cells in a tolerant state, thus preventing NKT cell overactivation after partial hepatectomy [42]. Moreover, circulating macrophages could also interact with liver endothelial cells, and this interaction was crucial for vascular growth and liver regeneration [48]. In addition, Kupffer cells were also required for the proliferation of liver progenitor cells that were able to differentiate into hepatocytes in a CDE diet-mediated liver injury and regeneration model [49].

Interestingly, in addition to hepatic macrophages, macrophages from other tissues could also be functional during liver regeneration. Recently, it is reported that a subset of F4/80hiGATA6+ macrophages in the peritoneal cavity could be rapidly recruited into the liver via the mesothelium and have pivotal reparative ability during liver regeneration [50].

### NK cells in liver regeneration

NK cells are another subset of innate immune cells that can kill invaded pathogens and transformed cells in different tissues. Through a sophisticated repertoire of activating and inhibitory receptors, their cytotoxicity activities are controlled delicately [51, 52]. It was demonstrated that bone marrow was the main development site for NK cells, providing various stimuli to guarantee its differentiation [53]. In mice, expressions of CD27 and CD11b define four sequential developmental stages of NK cells: CD11blowCD27low represents the most immature NK cells, then comes CD11blowCD27high, and then CD11bhighCD27high, and at last comes the most mature CD11bhighCD27low NK cells [54]. Among these NK cells, CD11bhighCD27high NK cells produce the largest amount of cytokines and are most cytotoxic [55], whereas the final step CD11bhighCD27low NK cells express the inhibitory receptor KLRG1 and show decreased effector functions and proliferation ability [56].

Apart from macrophages, NK cells represent another important component of the innate immune system in the liver [3]. NK cells constitute 30%~50% of the intrahepatic lymphocytes in humans, and 10%~15% in mice [57]. They are one of the most important cells in the control of bacterial and viral infection in the liver, such as HBV, HCV, murine cytomegalovirus, and Listeria monocytogenes infection [57, 58]. Liver NK cells are unique, because there is a subset of CD49a+DX5- liver-resident NK cells exerting memory responses in a contact hypersensitivity model [59]. Accumulating evidence suggests that NK cells are increased and activated in the liver after partial hepatectomy. In contrast to macrophages, liver NK cells inhibit rather than promote liver regeneration, and this inhibitory effect is likely mediated through the secretion of IFN-γ. The most convincing evidence for this is that liver regeneration is impaired when NK cells were activated by Poly I:C, and NK cell depletion enhanced liver regeneration rate. Moreover, liver regeneration was enhanced in IFN-γ deficient mice [60, 61]. The retarding effect of IFN-γ on liver regeneration was thought to be mediated by anti-proliferative proteins such as STAT1, IRF-1, and p21cip1/waf1 in the hepatocytes [60]. This function of NK cells in liver regeneration was recently supported by a study that TIGIT, which was a co-inhibitory receptor on NK cells, was involved in liver regeneration. TIGIT expression level was selectively up-regulated on liver NK cells after partial hepatectomy, and its deficiency promoted the activation of NK cells, ultimately hampering liver regeneration through TIGIT-PVR interaction [62].

### NKT cells in liver regeneration

NKT cells are a unique subset of T cells which are CD1d-restricted and lipid-antigen reactive [63]. In the liver, NKT cells have been shown to play a pathogenic role in ischemia reperfusion injury, nonalcoholic fatty liver disease, con-A induced hepatitis, primary biliary cholangitis, and so on [64–67]. Interestingly, NKT cells could produce both type I and type II cytokines, making them both pathogenic and protective. For instance, they are beneficial in acetaminophen-mediated acute liver injury, and they can also limit inflammatory cytokine secretion from macrophages in a CCl4 mediated acute liver injury model [68, 69]. Similar with NK cells, NKT cell number also increases in the regenerating liver. However, NKT cells may not be so potent as NK cells during liver regeneration, which was indicated by the fact that CD1d-/- or Jα281-/- mice showed similar regenerating rate compared with WT mice after partial hepatectomy [60, 70]. Nevertheless, NKT activation clearly impeded liver regeneration, which was also involved in IFN-γ mediated STAT1 signaling. This was strengthened by a finding that ampicillin-sensitive commensal bacteria depletion would activate liver NKT cells in an IL-12 dependent way and impair liver regeneration after partial hepatectomy [42]. Thus, it is likely that normal NKT cell biology was dispensable for liver regeneration, but activation of NKT cell by context-dependent stimuli would obviously impede liver regeneration after partial hepatectomy.

γδT cells in liver regeneration

Apart from macrophages, NK cells, and NKT cells, the liver is also selectively enriched in a special subset of T cells, namely γδT cells, which constitute about 15%~25% of the liver T cells. Most γδT cells are developed from the fetal thymus, from a common precursor of both αβT cells and γδT cells. Unlike αβT cells, a small proportion of γδT cells also generated in intestinal epithelium during early weeks of life [71]. To recognize antigens, αβT cells use T cell receptor (TCR) bound to CD3 to interact with the major histocompatibility complex on antigen-presenting cells, whereas γδT cells do not require such interaction [72]. Thus, γδT cells mainly sense early environmental signals to initiate local immno-surveilance, whereas the activation of αβT cells is relatively late [73]. In addition, even though γδT cells and αβT cells are both CD3 positive, most of γδT cells do not express CD4 and CD8 [73]. Like other cell subsets, γδT cells may be protective or pathogenic, and are also involved in various kinds of liver diseases, ranging from acute liver injury to liver cancer to chronic liver infection [6, 74]. It is observed that γδT cells are also activated and increased in number after partial hepatectomy [75]. Even though it had been reported earlier that IL-17A is crucial for normal liver regeneration [76], only in 2014 did Rao et al. found that it was γδT cell-derived IL-17A that exerted the regeneration-promoting function. They found that these IL-17A-secreting γδT cells were able to induce the production of IL-6 from antigen-presenting cells, and at the same time inhibited the secretion of IFN-γ from NKT cells, which meant that they could be both directly mitogenic for hepatocytes and promoted a regenerative-contributing phenotype in hepatic lymphocytes [75].

### Dendritic cells in liver regeneration

DCs are sparsely distributed within the liver. Usually, DCs in multiple tissues can be identified as CD45+ cells with high expression of MHCII and CD11c. However, to exclude other hematopoietic cell types when identifying DCs is very important, since DCs could also express some markers that are frequently expressed by other cells, such as macrophages and B cells [77]. In the liver, DCs can be divided into two subsets under steady state, namely plasmacytoid DCs (pDCs) and classical DCs (cDCs). pDCs express relatively lower levels of MHCII and thus have a limited ability to capture and present antigens, whereas cDCs express very high levels of MHCII and are very professional antigen-presenting cells [78]. Even though they are relatively rare in number, DCs are of great importance of liver immune regulation, such as liver fibrosis, alcoholic liver injury, as well as liver cancer [77–81]. In response to partial hepatectomy, liver DC number increased dramatically, indicating these cells were also functional during liver regeneration process [82]. Moreover, it was found that the partial hepatectomy-stimulated DC facilitated IL-10 production while inhibited IFN-γ production of T cells, and they could also enhanced estrogen receptor expression, thus promoting liver regeneration rate [82]. In a later study, researchers found that DCs were also capable of secreting TNF-α, and the reduced TNF-α production level would compromise liver regeneration rate [83].

### Eosinophils in liver regeneration

Eosinophils are myeloid derived cells. Upon activation, these cells are high granulated and will secrete cytokines, cytotoxic granule proteins, as well as enzymes and lipid mediators to kill pathogens or host cells [84]. These cells could be involved in a variety of pathogenic processes, such as asthma, allergy, and helminthic infection [84–88]. In the liver, these cells could be associated with transplantation rejection [89, 90], drug induced liver injury [91], liver fibrosis [92], and viral induced hepatitis [93, 94]. Of note, eosinophils also actively participate in the process of liver regeneration. In models of partial hepatectomy and toxin treatment, the number of eosinophils increased significantly. Also, eosinophil-lacking mice showed compromised regeneration rate of the liver. Later, it was found that eosinophil-derived IL-4 was the central factor promoting the proliferation of quiescent hepatocytes [95].

### Innate lymphoid cells in liver regeneration

The innate lymphoid cells (ILCs) are the most recently found members of the innate immune system and have been investigated intensely over the past six years. According to their specific surface markers, transcriptional factors, and the effector cytokines they secrete, the ILCs could be divided into three distinct subsets, namely group 1 ILCs (ILC1s), ILC2s, and ILC3s. All ILCs are developed from a common lymphoid progenitor, but lack the specific markers of other immune cell subsets. Moreover, unlike T cells and B cells, ILCs do not have antigen specificity because of their lack of antigen receptors [96, 97]. In multiple tissues of the body, ILCs can orchestrate homeostasis, inflammation and immunity through communicating with multiple cell types [98, 99]. In the liver, it was reported that CD49a+ ILC1s express high levels of NKG2A, thus limited the recruitment of peripheral NK cells, making the liver as a tolerogenic site during various kinds of viral infections [100, 101]. Moreover, it was reported that in response to IL-33, liver ILC2s were activated and secreted considerable amount of IL-13, leading to hepatic stellate cell activation and ultimately liver fibrosis [102]; and this circuit of signaling could also improve biliary repair to promote biliary carcinogenesis [103]. However, the functions of ILCs during the process of liver regeneration are largely unexplored. It was reported that liver specific IL-22 overexpression accelerated liver regeneration, yet the underlying mechanism was elusive [104]. Recently, it has been reported that ILC1s are indispensable for efficient liver regeneration. In this process, IL-22 is secreted by ILC1s in response to extracellular ATP signaling, and is identified as the critical mediator of liver regeneration after partial hepatectomy [105].

## THE ADAPTIVE IMMUNE SYSTEM AND LIVER REGENERATION

The adaptive immune responses are consisted of two forms: the humoral immunity, mediated by B cell-produced antibodies, and the cellular immunity, which is mediated by T cells [10]. Within the liver, the adaptive immune system is indispensable in a number of physiological and pathological processes, ranging from tolerance maintenance [106, 107], autoimmune diseases [107–110], tumors [111, 112], bacterial infection [50], viral infection [113–115], transplant rejection [116–118], obesity [119, 120], fibrosis [121–123], acute injuries [124–128], and so on. Nevertheless, the function of the adaptive immune system in the process of liver regeneration is rarely explored. However, this is not to say that the function of the adaptive immune system can be neglected. It was reported that T cell deficiency would greatly compromise the ability of liver regeneration and increase the mortality rate in mice after partial hepatectomy, indicating that T cells were critically indispensable for normal liver regeneration. The mechanism in this process was that T cell-secreted lymphotoxin could stimulate IL-6 production and signal transducer and activator of transcription 3 (STAT3) activation in the liver [129].

## CONCLUSIONS

**Figure 3:**
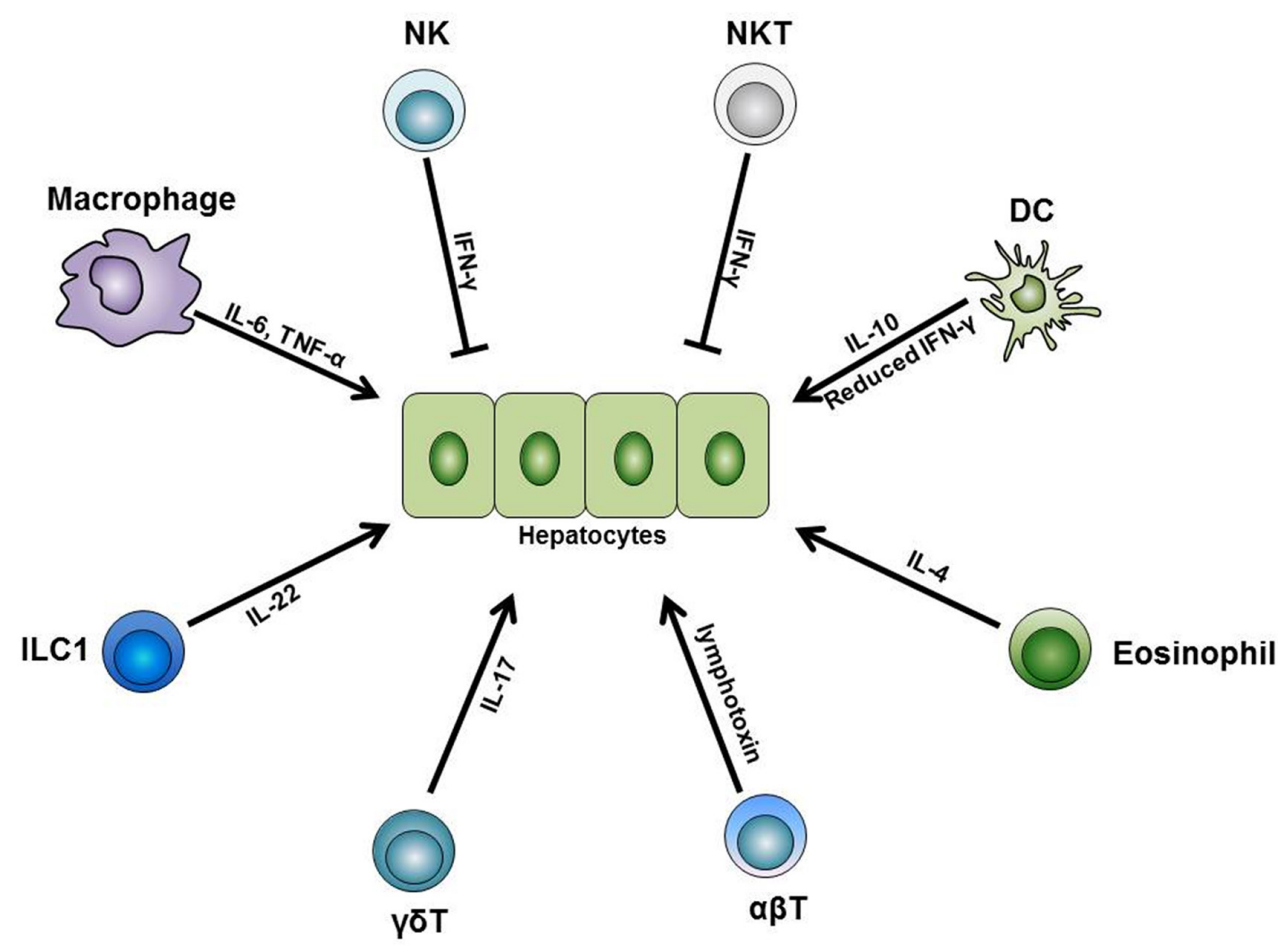
The role of immune cells during liver regeneration Different subsets of the innate and adaptive immune cells are indispensable for normal liver regeneration after partial hepatectomy. Among these cells, liver macrophages produce IL-6 and TNF-α and initiate the regeneration process after partial hepatectomy. Besides, liver DCs upregulate their IL-10 expression level while downregulate their IFN-γ level, thus facilitate liver regeneration. In addition, liver eosinophil-derived IL-4 also promotes the regeneration process. Furthermore, γδT cell-derived IL-17 and ILC1-derived IL-22 are both necessary for normal regeneration. On the other side, NK and NKT cells play inhibitory roles in liver regeneration, and this is mainly dependent on the IFN-γ they secrete. Besides these innate immune cells, conventional αβT cells can secrete lymphotoxin and stimulate liver regeneration. Abbreviations: TNF-α, tumor necrosis factor-α; IFN-γ, interferon-γ; ILC, innate lymphoid cell.

Liver regeneration is a complicate process that involves the cooperation of various immune cells (see Figure 3). Despite a great number of studies have been focusing on elucidating the mechanisms of the immune system in liver regeneration, many questions still need to be addressed. First, most studies concentrate on one specific cell subset, but further studies would be needed to investigate the cell-cell interactions in the liver cell network. Second, from a clinical perspective, investigators need to distinguish the differences between animal models and human cases, and more relevant and appropriate studies are needed to explore the regeneration process of clinical cases, such as toxins, tumors, ischemia reperfusion, or liver transplantation cases. In addition, considering the potential application of regenerative medicine, investigations concerning the transplantation or activation of intrahepatic stem cells are potentially promising for novel treatment of liver diseases.

## Abbreviations

NK: natural killer
NKT: natural killer T
DC: dendritic cell
γδT cell: gamma delta T cell
CD: Cluster of Differentiation
M-CSF: macrophage colony stimulating factor
ILC: innate lymphoid cell
STAT3: signal transducer and activator of transcription 3
IFN-γ: interferon-γ
TNF-α: tumor necrosis factor-α
IL-6: interlukin-6
SCF: stem cell factor
OSM: Oncostatin M
HGF: hepatocyte growth factor
EGF: epidermal growth factor
HB-EGF: heparin-binding epidermal growth factor
TGF: transforming growth factor
SOCS3: suppressor of cytokine signaling 3
MCP-1: monocyte chemoattractant protein-1
ICAM-1: intercellular adhesion molecule 1.

## References

[1] Stapleton GN, Hickman R, Terblanche J (1998). Blood supply of the right and left hepatic ducts. Brit J Surg.

[2] Kopka L, Rodenwaldt J, Vosshenrich R, Fischer U, Renner B, Lorf T, Graessner J, Ringe B, Grabbe E (1999). Hepatic blood supply: Comparison of optimized dual phase contrast-enhanced three-dimensional MR angiography and digital subtraction angiography. Radiology.

[3] Jenne CN, Kubes P (2013). Immune surveillance by the liver. Nature immunology.

[4] Wiest R, Lawson M, Geuking M (2014). Pathological bacterial translocation in liver cirrhosis. J Hepatol.

[5] Hackstein CP, Assmus LM, Welz M, Klein S, Schwandt T, Schultze J, Forster I, Gondorf F, Beyer M, Kroy D, Kurts C, Trebicka J, Kastenmuller W, Knolle PA, Abdullah Z (2016). Gut microbial translocation corrupts myeloid cell function to control bacterial infection during liver cirrhosis. Gut.

[6] Doherty DG (2016). Immunity, tolerance and autoimmunity in the liver: A comprehensive review. Journal of autoimmunity.

[7] Liaskou E, Wilson DV, Oo YH (2012). Innate Immune Cells in Liver Inflammation. Mediat Inflamm.

[8] Gao B, Jeong WI, Tian Z (2008). Liver: An organ with predominant innate immunity. Hepatology.

[9] Crispe IN (2009). The Liver as a Lymphoid Organ. Annu Rev Immunol.

[10] Shuai Z, Leung MW, He X, Zhang W, Yang G, Leung PS, Eric Gershwin M (2016). Adaptive immunity in the liver. Cellular & molecular immunology.

[11] Michalopoulos GK (2007). Liver regeneration. Journal of cellular physiology.

[12] Fausto N, Campbell JS, Riehle KJ (2006). Liver regeneration. Hepatology.

[13] Taub R (2004). Liver regeneration: from myth to mechanism. Nature reviews Molecular cell biology.

[14] Morrison JI, Loof S, He PP, Simon A (2006). Salamander limb regeneration involves the activation of a multipotent skeletal muscle satellite cell population. J Cell Biol.

[15] Volkman A, Chang NC, Strausbauch PH, Morahan PS (1983). Differential-Effects of Chronic Monocyte Depletion on Macrophage Populations. Lab Invest.

[16] Epelman S, Lavine KJ, Randolph GJ (2014). Origin and Functions of Tissue Macrophages. Immunity.

[17] Perdiguero EG, Geissmann F (2016). The development and maintenance of resident macrophages. Nature immunology.

[18] Schulz C, E Gomez Perdiguero, Chorro L, Szabo-Rogers H, Cagnard N, Kierdorf K, Prinz M, Wu B, Jacobsen SE, Pollard JW, Frampton J, Liu KJ, Geissmann F (2012). A lineage of myeloid cells independent of Myb and hematopoietic stem cells. Science.

[19] Klein I, Cornejo JC, Polakos NK, John B, Wuensch SA, Topham DJ, Pierce RH, Crispe IN (2007). Kupffer cell heterogeneity: functional properties of bone marrow derived and sessile hepatic macrophages. Blood.

[20] Zigmond E, Samia-Grinberg S, Pasmanik-Chor M, Brazowski E, Shibolet O, Halpern Z, Varol C (2014). Infiltrating monocyte-derived macrophages and resident kupffer cells display different ontogeny and functions in acute liver injury. J Immunol.

[21] Tacke F, Zimmermann HW (2014). Macrophage heterogeneity in liver injury and fibrosis. Journal of hepatology.

[22] Davies LC, Rosas M, Jenkins SJ, Liao CT, Scurr MJ, Brombacher F, Fraser DJ, Allen JE, Jones SA, Taylor PR (2013). Distinct bone marrow-derived and tissue-resident macrophage lineages proliferate at key stages during inflammation. Nature communications.

[23] Epelman S, Lavine KJ, Beaudin AE, Sojka DK, Carrero JA, Calderon B, Brija T, Gautier EL, Ivanov S, Satpathy AT, Schilling JD, Schwendener R, Sergin I, Razani B, Forsberg EC, Yokoyama WM (2014). Embryonic and adult-derived resident cardiac macrophages are maintained through distinct mechanisms at steady state and during inflammation. Immunity.

[24] Lavine KJ, Epelman S, Uchida K, Weber KJ, Nichols CG, Schilling JD, Ornitz DM, Randolph GJ, Mann DL (2014). Distinct macrophage lineages contribute to disparate patterns of cardiac recovery and remodeling in the neonatal and adult heart. Proceedings of the National Academy of Sciences of the United States of America.

[25] Martinez FO, Gordon S (2014). The M1 and M2 paradigm of macrophage activation: time for reassessment. F1000prime reports.

[26] Martinez FO, Sica A, Mantovani A, Locati M (2008). Macrophage activation and polarization. Frontiers in bioscience.

[27] Mantovani A, Sica A, Locati M (2005). Macrophage polarization comes of age. Immunity.

[28] Murray PJ, Allen JE, Biswas SK, Fisher EA, Gilroy DW, Goerdt S, Gordon S, Hamilton JA, Ivashkiv LB, Lawrence T, Locati M, Mantovani A, Martinez FO, Mege JL, Mosser DM, Natoli G (2014). Macrophage activation and polarization: nomenclature and experimental guidelines. Immunity.

[29] Gordon S, Martinez FO (2010). Alternative activation of macrophages: mechanism and functions. Immunity.

[30] Sica A, Erreni M, Allavena P, Porta C (2015). Macrophage polarization in pathology. Cellular and molecular life sciences.

[31] Sica A, Invernizzi P, Mantovani A (2014). Macrophage plasticity and polarization in liver homeostasis and pathology. Hepatology.

[32] Bilzer M, Roggel F, Gerbes AL (2006). Role of Kupffer cells in host defense and liver disease. Liver international : official journal of the International Association for the Study of the Liver.

[33] Abshagen K, Eipel C, Kalff JC, Menger MD, Vollmar B (2007). Loss of NF-kappa B activation in Kupffer cell-depleted mice impairs liver regeneration after partial hepatectomy. Am J Physiol-Gastr L.

[34] Nishiyama K, Nakashima H, Ikarashi M, Kinoshita M, Nakashima M, Aosasa S, Seki S, Yamamoto J (2015). Mouse CD11b(+)Kupffer Cells Recruited from Bone Marrow Accelerate Liver Regeneration after Partial Hepatectomy. Plos One.

[35] Shiratori Y, Hongo S, Hikiba Y, Ohmura K, Nagura T, Okano K, Kamii K, Tanaka T, Komatsu Y, Ochiai T, Tsubouchi H, Omata M (1996). Role of macrophages in regeneration of liver. Digestive diseases and sciences.

[36] Takeishi T, Hirano K, Kobayashi T, Hasegawa G, Hatakeyama K, Naito M (1999). The role of Kupffer cells in liver regeneration. Archives of histology and cytology.

[37] Yamada Y, Kirillova I, Peschon JJ, Fausto N (1997). Initiation of liver growth by tumor necrosis factor: deficient liver regeneration in mice lacking type I tumor necrosis factor receptor. Proceedings of the National Academy of Sciences of the United States of America.

[38] Cressman DE, Greenbaum LE, DeAngelis RA, Ciliberto G, Furth EE, Poli V, Taub R (1996). Liver failure and defective hepatocyte regeneration in interleukin-6-deficient mice. Science.

[39] Aldeguer X, Debonera F, Shaked A, Krasinkas AM, Gelman AE, Que XG, Zamir GA, Hiroyasu S, Kovalovich KK, Taub R, Olthoff KM (2002). Interleukin-6 from intrahepatic cells of bone marrow origin is required for normal murine liver regeneration. Hepatology.

[40] Selzner N, Selzner M, Odermatt B, Tian YH, N Van Rooijen, Clavien PA (2003). ICAM-1 triggers liver regeneration through leukocyte recruitment and Kupffer cell-dependent release of TNF-alpha/IL-6 in mice. Gastroenterology.

[41] Yang K, Du CY, Cheng Y, Li Y, Gong JP, Liu ZJ (2013). Augmenter of liver regeneration promotes hepatic regeneration depending on the integrity of Kupffer cell in rat small-for-size liver transplantation. J Surg Res.

[42] Song W, Mu H, Wu J, Liao M, Zhu H, Zheng L, He X, Niu B, Zhai Y, Bai C, Lei A, Li G, Hua J (2015). miR-544 regulates dairy goat male germline stem cell self-renewal via targeting PLZF. J Cell Biochem.

[43] Amemiya H, Kono H, Fujii H (2011). Liver Regeneration is Impaired in Macrophage Colony Stimulating Factor Deficient Mice After Partial Hepatectomy: The Role of M-CSF-Induced Macrophages. J Surg Res.

[44] Strey CW, Markiewski M, Mastellos D, Tudoran R, Spruce LA, Greenbaum LE, Lambris JD (2003). The proinflammatory mediators C3a and C5a are essential for liver regeneration. J Exp Med.

[45] Seki E, Tsutsui H, Iimuro Y, Naka T, Son G, Akira S, Kishimoto T, Nakanishi K, Fujimoto J (2005). Contribution of Toll-like receptor/myeloid differentiation factor 88 signaling to murine liver regeneration. Hepatology.

[46] Cornell RP (1985). Gut-derived endotoxin elicits hepatotrophic factor secretion for liver regeneration. The American journal of physiology.

[47] Wen YK, Feng DC, Wu HL, Liu WJ, Li HJ, Wang F, Xia Q, Gao WQ, Kong XN (2015). Defective Initiation of Liver Regeneration in Osteopontin-Deficient Mice after Partial Hepatectomy due to Insufficient Activation of IL-6/Stat3 Pathway. Int J Biol Sci.

[48] Melgar-Lesmes P, Edelman ER (2015). Monocyte-endothelial cell interactions in the regulation of vascular sprouting and liver regeneration in mouse. Journal of hepatology.

[49] Elsegood CL, Chan CW, Degli-Esposti MA, Wikstrom ME, Domenichini A, Lazarus K, van Rooijen N, Ganss R, Olynyk JK, Yeoh GCT (2015). Kupffer cell-monocyte communication is essential for initiating murine liver progenitor cell-mediated liver regeneration. Hepatology.

[50] Wang J, Kubes P (2016). A Reservoir of Mature Cavity Macrophages that Can Rapidly Invade Visceral Organs to Affect Tissue Repair. Cell.

[51] Lanier LL (2005). NK cell recognition. Annu Rev Immunol.

[52] Bernardini G, Benigni G, Antonangeli F, Ponzetta A, Santoni A (2014). Multiple levels of chemokine receptor regulation in the control of mouse natural killer cell development. Front Immunol.

[53] Di Santo JP (2006). Natural killer cell developmental pathways: A question of balance. Annu Rev Immunol.

[54] Chiossone L, Chaix J, Fuseri N, Roth C, Vivier E, Walzer T (2009). Maturation of mouse NK cells is a 4-stage developmental program. Blood.

[55] Hayakawa Y, Smyth MJ (2006). CD27 dissects mature NK cells into two subsets with distinct responsiveness and migratory capacity. Journal of Immunology.

[56] Huntington ND, Tabarias H, Fairfax K, Brady J, Hayakawa Y, Degli-Esposti MA, Smyth MJ, Tarlinton DM, Nutt SL (2007). NK cell maturation and peripheral homeostasis is associated with KLRG1 up-regulation. the Journal of Immunology.

[57] Sun HY, Sun C, Tian ZG, Xiao WH (2013). NK cells in immunotolerant organs. Cell Mol Immunol.

[58] Horowitz A, Stegmann KA, Riley EM (2012). Activation of Natural Killer cells during microbial infections. Front Immunol.

[59] Peng H, Jiang X, Chen Y, Sojka DK, Wei H, Gao X, Sun R, Yokoyama WM, Tian Z (2013). Liver-resident NK cells confer adaptive immunity in skin-contact inflammation. The Journal of clinical investigation.

[60] Sun R, Gao B (2004). Negative regulation of liver regeneration by innate immunity (natural killer cells/interferon-gamma). Gastroenterology.

[61] Vujanovic NL, Polimeno L, Azzarone A, Francavilla A, Chambers WH, Starzl TE, Herberman RB, Whiteside TL (1995). Changes of liver-resident NK cells during liver regeneration in rats. J Immunol.

[62] Bi JC, Zheng XD, Chen YY, Wei HM, Sun R, Tian ZG (2014). TIGIT Safeguards Liver Regeneration Through Regulating Natural Killer Cell-Hepatocyte Crosstalk. Hepatology.

[63] Godfrey DI, Stankovic S, Baxter AG (2010). Raising the NKT cell family. Nature immunology.

[64] Wallace KL, Marshall MA, Ramos SI, Lannigan JA, Field JJ, Strieter RM, Linden J (2009). NKT cells mediate pulmonary inflammation and dysfunction in murine sickle cell disease through production of IFN-gamma and CXCR3 chemokines. Blood.

[65] Halder RC, Aguilera C, Maricic I, Kumar V (2007). Type II NKT cell-mediated anergy induction in type I NKT cells prevents inflammatory liver disease. The Journal of clinical investigation.

[66] Schrumpf E, Tan C, Karlsen TH, Sponheim J, Bjorkstrom NK, Sundnes O, Alfsnes K, Kaser A, Jefferson DM, Ueno Y, Eide TJ, Haraldsen G, Zeissig S, Exley MA, Blumberg RS, Melum E (2015). The biliary epithelium presents antigens to and activates natural killer T cells. Hepatology.

[67] Bandyopadhyay K, Marrero I, Kumar V (2016). NKT cell subsets as key participants in liver physiology and pathology. Cell Mol Immunol.

[68] Martin-Murphy BV, Kominsky DJ, Orlicky DJ, Donohue TM, Ju C (2013). Increased susceptibility of natural killer T-cell-deficient mice to acetaminophen-induced liver injury. Hepatology.

[69] Kwon HJ, Won YS, Park O, Feng DC, Gao B (2014). Opposing Effects of Prednisolone Treatment on T/NKT Cell- and Hepatotoxin-Mediated Hepatitis in Mice. Hepatology.

[70] Hosoya S, Ikejima K, Arai K, Kon K, Yamashina S, Takeda K, Watanabe S (2011). Innate Immune Responses Involving Nk and Nkt Cells Promote Liver Regeneration after Partial Hepatectomy. Hepatology.

[71] Porcelli S, Morita CT, Brenner MB (1992). CD1b restricts the response of human CD4-8- T lymphocytes to a microbial antigen. Nature.

[72] Born WK, M Kemal Aydintug, O’Brien RL (2013). Diversity of gammadelta T-cell antigens. Cellular & molecular immunology.

[73] Marquez-Medina D, Salla-Fortuny J, Salud-Salvia A (2012). Role of gamma-delta T-cells in cancer: another opening door to immunotherapy. Clinical & translational oncology.

[74] Hammerich L, Tacke F (2014). Role of gamma-delta T cells in liver inflammation and fibrosis. World journal of gastrointestinal pathophysiology.

[75] Rao R, Graffeo CS, Gulati R, Jamal M, Narayan S, Zambirinis CP, Barilla R, Deutsch M, Greco SH, Ochi A, Tomkotter L, Blobstein R, Avanzi A, Tippens DM, Gelbstein Y, E Van Heerden (2014). Interleukin 17-Producing gamma delta T Cells Promote Hepatic Regeneration in Mice. Gastroenterology.

[76] Furuya S, Kono H, Hara M, Hirayama K, Tsuchiya M, Fujii H (2013). Interleukin-17A plays a pivotal role after partial hepatectomy in mice. J Surg Res.

[77] Merad M, Sathe P, Helft J, Miller J, Mortha A (2013). The dendritic cell lineage: ontogeny and function of dendritic cells and their subsets in the steady state and the inflamed setting. Annu Rev Immunol.

[78] Rahman AH, Aloman C (2013). Dendritic cells and liver fibrosis. Biochimica et biophysica acta.

[79] Almeda-Valdes P, NE Aguilar Olivos, Barranco-Fragoso B, Uribe M, Mendez-Sanchez N (2015). The Role of Dendritic Cells in Fibrosis Progression in Nonalcoholic Fatty Liver Disease. BioMed research international.

[80] Lukacs-Kornek V, Schuppan D (2013). Dendritic cells in liver injury and fibrosis: shortcomings and promises. J Hepatol.

[81] Tacke F, Yoneyama H (2013). From NAFLD to NASH to fibrosis to HCC: role of dendritic cell populations in the liver. Hepatology.

[82] Castellaneta A, A Di Leo, Francavilla R, Margiotta M, Barone M, Amoruso A, Troiani L, Thomson AW, Francavilla A (2006). Functional modification of CD11c(+) liver dendritic cells during liver regeneration after partial hepatectomy in mice. Hepatology.

[83] Wolf JH, Bhatti TR, Fouraschen S, Chakravorty S, Wang LQ, Kurian S, Salomon D, Olthoff KM, Hancock WW, Levine MH (2014). Heat shock protein 70 is required for optimal liver regeneration after partial hepatectomy in mice. Liver Transplant.

[84] Kita H (2011). Eosinophils: multifaceted biological properties and roles in health and disease. Immunological reviews.

[85] Venge P (2010). The eosinophil and airway remodelling in asthma. The clinical respiratory journal.

[86] Magalhaes K, Almeida PE, Atella G, Maya-Monteiro CM, Castro-Faria-Neto H, Pelajo-Machado M, Lenzi HL, Bozza MT, Bozza PT (2010). Schistosomal-derived lysophosphatidylcholine are involved in eosinophil activation and recruitment through Toll-like receptor-2-dependent mechanisms. The Journal of infectious diseases.

[87] Stolarski B, Kurowska-Stolarska M, Kewin P, Xu D, Liew FY (2010). IL-33 exacerbates eosinophil-mediated airway inflammation. J Immunol.

[88] Trivedi SG, Lloyd CM (2007). Eosinophils in the pathogenesis of allergic airways disease. Cellular and molecular life sciences.

[89] Wang GY, Li H, Wang GS, Zhang J, Chen GH (2012). Elevated eosinophil count is positively related with late acute cellular rejection after liver transplantation. J Gastroen Hepatol.

[90] Rodriguez-Peralvarez M, Germani G, Tsochatzis E, Rolando N, Luong TV, Dhillon AP, Thorburn D, O’Beirne J, Patch D, Burroughs AK (2012). Predicting severity and clinical course of acute rejection after liver transplantation using blood eosinophil count. Transplant international.

[91] Proctor WR, Chakraborty M, Chea LS, Morrison JC, Berkson JD, Semple K, Bourdi M, Pohl LR (2013). Eosinophils mediate the pathogenesis of halothane-induced liver injury in mice. Hepatology.

[92] Reiman RM, Thompson RW, Feng CG, Hari D, Knight R, Cheever AW, Rosenberg HF, Wynn TA (2006). Interleukin-5 (IL-5) augments the progression of liver fibrosis by regulating IL-13 activity. Infection and immunity.

[93] Tarantino G, Cabibi D, Camma C, Alessi N, Donatelli M, Petta S, Craxi A, Di Marco V (2008). Liver eosinophilic infiltrate is a significant finding in patients with chronic hepatitis C. J Viral Hepatitis.

[94] Martineosuna P, Espinoza CG, Cuellar ML, Cabrera GE, Silveira LH, Espinoa LR (1994). Eosinophilic Hepatitis - a New Feature of the Clinical Spectrum of the Clinical Spectrum of the Eosinophilia-Myalgia-Syndrome. Clin Rheumatol.

[95] Goh YPS, Henderson NC, Heredia JE, Eagle AR, Odegaard JI, Lehwald N, Nguyen KD, Sheppard D, Mukundan L, Locksley RM, Chawla A (2013). Eosinophils secrete IL-4 to facilitate liver regeneration. Proceedings of the National Academy of Sciences of the United States of America.

[96] Diefenbach A, Colonna M, Koyasu S (2014). Development, differentiation, and diversity of innate lymphoid cells. Immunity.

[97] Artis D, Spits H (2015). The biology of innate lymphoid cells. Nature.

[98] Spits H, Artis D, Colonna M, Diefenbach A, JP Di Santo, Eberl G, Koyasu S, Locksley RM, McKenzie ANJ, Mebius RE, Powrie F, Vivier E (2013). Innate lymphoid cells - a proposal for uniform nomenclature. Nat Rev Immunol.

[99] Spits H, Di Santo JP (2011). The expanding family of innate lymphoid cells: regulators and effectors of immunity and tissue remodeling. Nature immunology.

[100] Yang Z, Tang T, Wei X, Yang S, Tian Z (2015). Type 1 innate lymphoid cells contribute to the pathogenesis of chronic hepatitis B. Innate immunity.

[101] Krueger PD, Narayanan S, Surette FA, Brown MG, Sung SJ, Hahn YS (2016.). Murine liver-resident group 1 innate lymphoid cells regulate optimal priming of anti-viral CD8+ T cells. Journal of leukocyte biology.

[102] McHedlidze T, Waldner M, Zopf S, Walker J, Rankin AL, Schuchmann M, Voehringer D, McKenzie AN, Neurath MF, Pflanz S, Wirtz S (2013). Interleukin-33-dependent innate lymphoid cells mediate hepatic fibrosis. Immunity.

[103] Li J, Razumilava N, Gores GJ, Walters S, Mizuochi T, Mourya R, Bessho K, Wang YH, Glaser SS, Shivakumar P, Bezerra JA (2014). Biliary repair and carcinogenesis are mediated by IL-33-dependent cholangiocyte proliferation. The Journal of clinical investigation.

[104] Park O, Wang H, Weng HL, Feigenbaum L, Li H, Yin S, Ki SH, Yoo SH, Dooley S, Wang FS, Young HA, Gao B (2011). In Vivo Consequences of Liver-Specific Interleukin-22 Expression in Mice: Implications for Human Liver Disease Progression. Hepatology.

[105] Kudira R, Malinka T, Kohler A, Dosch M, de Aguero MG, Melin N, Haegele S, Starlinger P, Maharjan N, Saxena S, Keogh A, Stroka D, Candinas D, Beldi G (2016). P2×1-regulated IL-22 secretion by innate lymphoid cells is required for efficient liver regeneration. Hepatology.

[106] Tiegs G, Lohse AW (2010). Immune tolerance: What is unique about the liver. Journal of autoimmunity.

[107] Doherty DG (2016). Immunity, tolerance and autoimmunity in the liver: A comprehensive review. J Autoimmun.

[108] Lampinen M, Fredricsson A, Vessby J, Wanders A, Rorsman F, Carlson M (2016). Expression of the liver homing receptor CXCR3+on colonic CD8+T lymphocytes in patients with primary sclerosing cholangitis provides a possible link between colonic and biliary duct inflammation. J Crohns Colitis.

[109] Zhao SX, Wang RQ, Zhang YG, Du HJ, Nan YM (2015). Emperipolesis mediated by CD8+T cells correlated with biliary epithelia cell injury in primary biliary cirrhosis. Hepatology.

[110] Zhang J, Zhang W, Leung PS, Bowlus CL, Dhaliwal S, Coppel RL, Ansari AA, Yang GX, Wang J, Kenny TP, He XS, Mackay IR, Gershwin ME (2014). Ongoing activation of autoantigen-specific B cells in primary biliary cirrhosis. Hepatology.

[111] Pedroza-Gonzalez A, Kwekkeboom J, Tjwa ET, Polak WG, Grunhagen DJ, IJzermans JNM, Janssen HLA, Sprengers D (2013). Abrogation of Tumor-Associated Immunosuppression by Targeting Tumor-Infiltrating Regulatory T-Cells Restores Impaired T Cell Responses in Patients with Liver Cancer. J Hepatol.

[112] Pedroza-Gonzalez A, Verhoef C, Ijzermans JN, Peppelenbosch M, Kwekkeboom J, Janssen HL, Sprengers D (2012). Tumor-Infiltrating Regulatory T Cells Favor Tumor Development by Suppressing Local Tumor-Specific T Cell Responses in Primary and Metastatic Liver Cancer. J Hepatol.

[113] Dolina JS, Braciale TJ, Hahn YS (2014). Liver-primed CD8+ T cells suppress antiviral adaptive immunity through galectin-9-independent T-cell immunoglobulin and mucin 3 engagement of high-mobility group box 1 in mice. Hepatology.

[114] Minagawa M, Kawamura H, Liu Z, Govindarajan S, Dennert G (2005). Suppression of adenoviral gene expression in the liver: role of innate vs adaptive immunity and their cell lysis mechanisms. Liver international.

[115] Fan HB, Zhu YF, Chen AS, Zhou MX, Yan FM, Ma XJ, Zhou H (2009). B-cell clonality in the liver of hepatitis C virus-infected patients. World journal of gastroenterology.

[116] Shalev I, Selzner N, Shyu W, Grant D, Levy G (2012). Role of regulatory T cells in the promotion of transplant tolerance. Liver transplantation.

[117] Forbes SJ, Gupta S, Dhawan A (2015). Cell therapy for liver disease: From liver transplantation to cell factory. J Hepatol.

[118] Ningappa M, Ashokkumar C, Higgs BW, Sun Q, Jaffe R, Mazariegos G, Li D, Weeks DE, Subramaniam S, Ferrell R, Hakonarson H, Sindhi R (2016). Enhanced B Cell Alloantigen Presentation and Its Epigenetic Dysregulation in Liver Transplant Rejection. American journal of transplantation.

[119] Tada F, Abe M, Kawasaki K, Miyake T, Shiyi C, Hiasa Y, Matsuura B, Onji M (2013). B cell activating factor in obesity is regulated by oxidative stress in adipocytes. J Clin Biochem Nutr.

[120] Fabbrini E, Cella M, Mccartney SA, Fuchs A, Abumrad NA, Pietka TA, Chen ZJ, Finck BN, Han DH, Magkos F, Conte C, Bradley D, Fraterrigo G, Eagon JC, Patterson BW, Colonna M (2013). Association Between Specific Adipose Tissue CD4(+) T-Cell Populations and Insulin Resistance in Obese Individuals. Gastroenterology.

[121] Glassner A, Eisenhardt M, Kokordelis P, Kramer B, Wolter F, Nischalke HD, Boesecke C, Sauerbruch T, Rockstroh JK, Spengler U, Nattermann J (2013). Impaired CD4(+) T cell stimulation of NK cell anti-fibrotic activity may contribute to accelerated liver fibrosis progression in HIV/HCV patients. J Hepatol.

[122] Holt AP, Stamataki Z, Adams DH (2006). Attenuated liver fibrosis in the absence of B cells. Hepatology.

[123] Safadi R, Ohta M, Alvarez CE, Fiel MI, Bansal M, Mehal WZ, Friedman SL (2004). Immune stimulation of hepatic fibrogenesis by CD8 cells and attenuation by transgenic interleukin-10 from hepatocytes. Gastroenterology.

[124] Gantner F, Leist M, Lohse AW, Germann PG, Tiegs G (1995). Concanavalin A-induced T-cell-mediated hepatic injury in mice: the role of tumor necrosis factor. Hepatology.

[125] Wang X, Sun R, Chen Y, Lian ZX, Wei H, Tian Z (2015). Regulatory T cells ameliorate acetaminophen-induced immune-mediated liver injury. International immunopharmacology.

[126] Boag SE, Das R, Shmeleva EV, Bagnall A, Egred M, Howard N, Bennaceur K, Zaman A, Keavney B, Spyridopoulos I (2015). T lymphocytes and fractalkine contribute to myocardial ischemia/reperfusion injury in patients. The Journal of clinical investigation.

[127] Linfert D, Chowdhry T, Rabb H (2009). Lymphocytes and ischemia-reperfusion injury. Transplantation reviews.

[128] Ke B, Shen XD, Kamo N, Ji H, Yue S, Gao F, Busuttil RW, Kupiec-Weglinski JW (2013). beta-catenin regulates innate and adaptive immunity in mouse liver ischemia-reperfusion injury. Hepatology.

[129] Tumanov AV, Koroleva EP, Christiansen PA, Khan MA, Ruddy MJ, Burnette B, Papa S, Franzoso G, Nedospasov SA, Fu YX, Anders RA (2009). T cell-derived lymphotoxin regulates liver regeneration. Gastroenterology.

[130] Czaja MJ, Geerts A, Xu J, Schmiedeberg P, Ju Y (1994). Monocyte chemoattractant protein 1 (MCP-1) expression occurs in toxic rat liver injury and human liver disease. Journal of leukocyte biology.

[131] Marra F, DeFranco R, Grappone C, Milani S, Pastacaldi S, Pinzani M, Romanelli RG, Laffi G, Gentilini P (1998). Increased expression of monocyte chemotactic protein-1 during active hepatic fibrogenesis: correlation with monocyte infiltration. The American journal of pathology.

[132] Ding BS, Nolan DJ, Butler JM, James D, Babazadeh AO, Rosenwaks Z, Mittal V, Kobayashi H, Shido K, Lyden D, Sato TN, Rabbany SY, Rafii S (2010). Inductive angiocrine signals from sinusoidal endothelium are required for liver regeneration. Nature.

[133] Racanelli V, Rehermann B (2006). The liver as an immunological organ. Hepatology.

